# Pruritus Ani

**Published:** 1905-10

**Authors:** Charles J. Drueck

**Affiliations:** Chicago, Ill., Professor of Rectal Diseases at Harvey Medical College; Professor of Rectal Diseases and Clinical Surgery at National Medical University; Rectal Surgeon to the Peoples’ Hospital and Attending Surgeon to the National Emergency, and the Lakeside Hospitals, Chicago


### Pruritus Ani.

By CHARLES J. DRUECK, M. D., Chicago, Ill.,

Professor of Rectal Diseases at Harvey Medical College; Professor of Rectal Diseases and Clinical Surgery at National Medical University; Rectal Surgeon to the Peoples' Hospital and Attending Surgeon to the National Emergency, and the Lakeside Hospitals, Chicago.

[From *The Medical Standard*, September, 1905.]

PRURITUS ani is the name applied to any itching about the anus regardless of the cause, and may be a direct symptom of some local rectal disorder or a reflex manifestation of some other diseased pelvic or abdominal organ or even the sign of serious systemic disturbance. The character or degree of itching may vary and includes all varieties of eczema. Sometimes the cause is discernable, while again, it is quite obscure, and in some instances there seems to be no apparent source. The general practitioner often considers it a trifling ailment, although in old and aggravated cases it proves to be intractable and renders the patient's life almost unbearable.

It affects all classes of men and women, for it is found in all occupations and social conditions of life and occurs at any age, but is more common in males and about middle age. The itching is usually constant with exacerbations, especially at night, when the patient's body becomes warm in bed and is not limited to the anus, but radiates down the limbs, over the buttocks and across the perineum to the urinary organs. Scratching and rubbing the parts gives only temporary relief and leaves after-effects such as bleeding, cracks, and fissure, which add to the annoyance. In the majority of cases, pruritus is simply a symptom of some other rectal or pelvic trouble and may therefore, refer to any neighboring organ, though it is usually due to hyperesthesia of the nerve filaments.

*Etiology.*—The causes are many and sometimes difficult to ascertain, but usually when found and removed the pruritus will be cured at once. Sometimes nothing can be found to explain the itching. Various local and constitutional conditions or habits are at times the source or occasionally neurotic affections, diabetes, portal congestion, syphilis, rheumatism, albuminuria, or a gouty tendency. Thread-worms are frequently the cause in children, but rarely in adults and can easily be found in the folds of the mucous membrane of the rectum, as short fibers resembling pieces of thread. Chronic prostatis, proctitis, impacted feces, chronic constipation and errors of diet are each at times the origin. Hemorrhoids, either internal or external, ulceration, a beginning fissure, prolapse, polytus, fistula in any variety may be underlying causes. In women it occurs with pruritus vulvæ, during menstruation and early during pregnancy or in the last few weeks of gestation, when the perineum is congested and edematous.

Certain articles of food often cause the pruritus, as, shellfish, salmon, venison, strawberries, coffee or the overuse of meats and spices. In fact, any rich diet or excess of alcohol or tobacco may produce it.

When a fistula is present the skin opening may be pin-point in size, yet the discharge as it evaporates leaves a crust which occasions the itching. So also do pediculi by their presence and excretions. Herpes, erythema in fleshy people from chafing and sweating of the buttocks in walking, and eczema either acute or chronic, are each occasionally the cause of pruritus. In eczema marginata, the spores may be found by the microscope in scales scraped off and moistened in glycerin. Incontinence of urine in either children or old people is frequently the source.

*Symptoms.*—The predominant symptoms is the unbearable itching which grows worse with warmth and rubbing of the parts. Scratching, which is sometimes controlled when the sufferer is awake, is unconscientiously performed when he is asleep, and causes bleeding and abrasions of the skin which add to the condition the next day.

In recent cases, there is often nothing to see or there may be a red, glistening, perhaps edematous condition of the parts, with a scanty serous secretion, sufficient to keep the skin moist. In old cases, the skin becomes thick, pale and parchment-like, sometimes even brownish, especially back of the anus, and toward the coccyx in the intergluteal space where sometimes the whole surface is eroded and fissured. In protracted cases, from prolonged insomnia and nervous wear, the patient's general health and nutrition fail and he becomes a hypochondriac.

*Diagnosis.*—Pruritus is easily diagnosticated from the patient's history and then corroborated by the examination. The patient in the lithotomy or Sims position affords easy inspection of the whole field. The presence of syphilis, tuberculosis and especially rheumatism as an underlying dyscrascia must always be investigated in obscure cases, because this fact alters the treatment.

*Prognosis.*—Without careful attention, the outlook is very poor. Periods of improvement are quickly followed by exacerbations worse than the preceding. The itching and pain, together with the loss of sleep, bring these sufferers to the verge of nervous prostration or insanity, if they have not already become opium wrecks. A patient told me that he had not had a day's peace or a comfortable night's sleep in a year. He could do no business, looked and felt as though he was in the last stage of phthisis and vowed unless I could help him, he would commit suicide. Under careful, persistent treatment you may look for a favorable future provided the cause can be removed. The patient, however, must be told that unless the cause is purely local the treatment requires time and perseverance on his part.

*Treatment.*—The treatment depends first upon the cause, for with that removed, the pruritus will usually disappear. Sometimes the cause is very trivial. I remember seeing a severe pruritus that had resisted treatment by two physicians, that was due to a few inverted hairs. These were removed, the follicles destroyed and sedative dusting powder used. The case healed very kindly and has since never bothered the patient, who is himself a physician. If there is no apparent local cause, examine the patient's general health and condition. When anemia or tuberculosis exists, alternatives and tonics like arsenic, iron, cod-liver oil and quinine are indicated, also nux vomica for its tonic effect, both systemic and intestinal. The tonic value of exercise must be remembered and its importance impressed upon the patient. The diet is to be looked into and excesses cut off, such as coffee, alcohol, tobacco, meats, spices, condiments and highly seasoned sauces. If the gastric digestion is impaired, pepsin, diatase or mineral acids may be needed. In the lower digestion, the bowel or liver may need attention. The genitourinary systems in both male and female patients must be given a thorough examination and the physician satisfied that there is nothing about these organs that is abnormal, which might maintain a reflex excitation.

The condition of the stool should be inquired into and if constipation exists, the bowel should be thoroughly emptied by a saline laxative or rhubarb, after which they should be moved daily. Aloes is contraindicated as a laxative because of its irritating effect on the rectum. The use of intestinal antiseptics such as salol, sulphocarbolates and ichthyol, five grains of the latter given night and morning on an empty stomach, is useful to clear up the autointoxication. Intestinal lavage with large quantities of weak alkaline solutions is of inestimable value in emptying the bowel and stimulating intestinal action (the details of lavage will be considered at length at another time in a paper on constipation and autointoxication, because of the lack of space in this article.)

Syphilis occasionally produces a pruritus and eczema about the anus, but a thorough course of mercury and the iodides will remove it. If due to lithemia, the salicylates and alkalies should be given freely. As the itching is always worse while in bed and when the patient is resting, the use of pajama night suits must be replaced by a loose-fitting cotton gown which does not touch the perineum. The pajamas, by fitting closely, increase the production of moisture and the chafing. Cotton sheets should also be worn on the bed and wool blankets substituted for heavy quilts, that a more even temperature may be maintained and therefore, less likeliness to sweat.

*Local Treatment.*—Locally, the treatment divides itself into cleanliness, keeping the parts dry, giving rest by preventing friction between the sides, and relieving sphincteric spasm. In conjunction with local applications, to be described later; I have made it an almost routine practice, whether the cause is evident or not, to gradually but thoroughly dilate the sphincter. There is always a hypersensitiveness of the anal mucous membrane, or an hypertrophy of the sphincter muscles, which are both relieved by this procedure. The dilatation is performed slowly and without an anesthetic. By frequent moderate stretchings I overcome the hyperesthesia and abnormal resistance, and thus relieve the constipation that occurs from prolonged retention of the feces. This also relieves the tenesmus that is set up and which keeps the skin under tension and helps to make the defecation painful. It has been advised to perform the dilatation under chloroform, but I do not employ that method, because whenever you dilate forcibly there is danger of incontinence resulting which will be more or less permanent. This mishap is never possible when the dilatation is performed by my method.

The local application of astringents and ointments, as acetate of lead or zinc, zinc oxide, carbolic acid, chloroform (a dram to the ounce of olive oil), bismuth or mercury may be tried, but with varying success, unless the predisposing cause is removed. Opium is to be used very advisedly because of the possibility of inducing the habit and it must be remembered that the secondary effect of the drug is to produce a general itching. I have found mutton suet and diachylon ointment preferable to other emollients. The following formula I have frequently used to give temporary relief while treating the underlying cause:

℞ Camphoræ,

Chloral hydratis .............................. aa dram 1
Ung. diachylon ................................ ounce 1

This may be applied two or three times daily after bathing the parts. Much immediate relief is obtained by brushing the surface with nitrate of silver, twenty grains to the ounce, or with Churchill tincture of iodine, twice a week. Both applications produce some temporary pain, but the relief afterward fully compensates for the suffering. When the pruritus is due to the thread-worms, injections of lime water or salt water are sufficient or in severe cases, an anthelmintic, as santonin. Occasionally, this procedure needs to be repeated.

*Importance of Cleanliness.*—Scrupulous cleanliness during the local toilet is imperative. The matter of toilet papers is important, for harsh papers or ordinary newspaper, on account of the printer's ink, may cause pruritus. A patient whom the writer saw presented a very obstinate case of pruritus that persistently recurred until it was discovered that the sufferer used for toilet paper, the paper which had been wrapped around oranges and lemons. When this cause was removed and a little local treatment given, the case healed and has never recurred. Where contact of the feces produces the pruritus, the ordinary use of toilet paper is not sufficient to remove the small particles and plegets of cotton moistened in warm water is much more satisfactory. The addition of sodium borate or bicarbonate to the water is often of value. When the pruritus is due to vaginal discharges, douches of one to two thousand mercuric chloride, or 2 per cent. lysol should be used twice daily. In infants, when due to lack of attention during an attack of diarrhea or to fermentation of urine about the parts, I have found picric acid, 10 per cent. in nitrate of mercury ointment, gives the best results.

Eczema in all its various forms is frequently a cause. If the skin is dry and scaly much benefit is derived from tar preparations. Bathe the parts with a solution of alcohol and tar-water or an ointment as:

| | | |
|---|---|---|
| ℞ | Picis liquidæ | drams 4 |
| | Ung. belladonnæ | drams 2 |
| | Ac. carbolici | m x |
| | Adeps lanæ | drams 2 |

Bathe the parts frequently in water as hot as can be borne and green soap, to remove the thickened scales and to deplete the local circulation. In exaggerated cases a solution of caustic potash, five grains to the ounce, may be used. A cloth may be used to sop the hot water on the parts, but do not allow any rubbing. This treatment on retiring will often insure a restful sleep for an otherwise tortured patient.

Eczematous Varities.—In the moist variety of eczema and in erythema, soothing applications, such as calomel, bismuth, boric acid, starch or zinc oxide powders, may be dusted on the parts and a piece of lint or muslin inserted between the buttocks. The following as an ointment has acted admirably in many of these cases:

| | | |
|---|---|---|
| ℞ | Thymol | grains 2 |
| | Pulv. zinc. stearat. | drams 4 |

When the patient is about to indulge in excessive exercise, work or walking, especially obese subjects, where he is likely to aggravate the condition, he should apply the above ointment before the effort and bathe the parts promptly afterward with cold water, being careful to dry them thoroughly. Frequent ablutions of cold water give much temporary relief in these cases. In fleshy individuals and others who perspire freely and are likely to chafe, carbolic acid solution ½ per cent. or potassium permangate 1-5000 is an admirable lotion to be used daily with the toilet.

In eczema marginata, thoroughly bathe the parts with warm water and soap, carefully dry with a soft cloth and then apply dilute sulphuric acid. It is painful for a short while but gives immediate relief. Tincture of iodine may be used.

In purely neurasthenic cases, local treatment is of no use and the patient is better off without it. Moral and hygienic instructions are much more needed and in some instances, "straight talk" as Morris (*British Medical Journal*) calls it, will do more than all the drugs. In severe cases, or when the pruritus is due to disease of the brain, spinal cord or their membranes, it may be necessary to give the patient a narcotic to enable him to sleep. Bromide or chloral is preferable to opium, as the later causes more itching the next day.

USE OF *x*-RAYS.—Recently, the Röntgen ray has been used in the treatment of pruritus and eczema about these parts, but the work in many instances has been done by enthusiasts and its value overestimated. In many cases, but not all, the *x*-ray acts almost magically, still there is always danger of possible burns even in the hands of an expert, and this danger is increased, if the treatment is continued over a long period of time. A fifteen-minute treatment every other day for two weeks ought to show results by relieving the itching and increasing the discoloration of the skin. A tube of low vacuum is held about ten inches from the patient and the surrounding parts are protected by lead sheet. No application or ointment containing metallic salts is to be used at any time during this treatment. Nothing is worse in the treatment of this trouble than the empirical use of any agent and it is folly to use the *x*-ray on every case without first finding the cause of the itching. The *x*-ray, however, will prove a valuable assistant if its use is limited to cases of unknown origin or those that prove themselves especially obstinate and where relief alone is desired. A cure cannot be expected, even with the use of the *x*-ray, unless the cause is removed. Should a cure occur, it will be only temporary, if the exciting cause is allowed to continue.

When the pruritus is due to proctitis, hemorrhoids, fissure ulceration, fistula, prolapse or polypus, and the patient refuses to submit to surgical treatment, or in senile, debilitated or hemorrhagic subjects, much relief may be given by the use of the following:

| | | |
|---|---|---|
| ℞ | Calomel | grains 30 |
| | Menthol | grains 10–20 |
| | Vaseline | ounce 1 |

Sig.—Apply after each bowel movement, after bathing the surface carefully and sopping dry.

If there is much pain, cocaine hydrochlorate, grains ten, may be substituted in the above prescription for the menthol (Mathews). When hemorrhoids are the cause, the use of unguentum Gallæ or tincture of iodine will relieve the itching. These formulæ, in a general way, have given me much satisfaction, but must be altered to suit each case.

Finally, there remains a class of cases in which the pruritus persists in spite of all therapeutic treatment and where there is no apparent cause, either local or systemic. In these instances the actual cautery applied lightly all over the irritated surface will sometimes effect a cure.

